# Quality of care assessment and improvement in aortic stenosis - rationale and design of a multicentre registry (IMPULSE)

**DOI:** 10.1186/s12872-016-0439-4

**Published:** 2017-01-05

**Authors:** Norbert Frey, Richard P. Steeds, Antonio Serra, Eberhard Schulz, Stephan Baldus, Matthias Lutz, Christiane Pohlmann, Jana Kurucova, Peter Bramlage, David Messika-Zeitoun

**Affiliations:** 1 Department of Cardiology and Angiology, University of Kiel, Kiel, Germany; 2 Queen Elizabeth Hospital and Institute of Cardiovascular Sciences, University of Birmingham, Birmingham, UK; 3 Hospital de Sant Pau, Cardiology Unit, University of Barcelona, Barcelona, Spain; 4 Cardiology Department I, University Clinic Mainz, Mainz, Germany; 5 Clinic for Cardiology, Angiology, and Pneumology and Intensive Care Medicine, Heart Center of the University Clinic Cologne, Cologne, Germany; 6 Institute for Pharmacology and Preventive Medicine, Cloppenburg, Germany; 7 Edwards Lifesciences, Prague, Czech Republic; 8 Department of Cardiology, Bichat Hospital, Paris, France

**Keywords:** Quality of care, Aortic stenosis, Transcatheter aortic valve implantation, Surgical aortic valve replacement, Facilitated data relay

## Abstract

**Background:**

Severe aortic stenosis (AS) is a common, serious valve disease in which no effective medical therapy is available and, if not treated by intervention, has a 5-year survival of only 40–60%. Despite the availability of guidelines supporting the effective use of surgical aortic valve replacement (SAVR) or transcatheter aortic valve implantation (TAVI) to treat the majority of these patients, adherence to these guidelines in clinical practice is still unsatisfactory. Several recent studies have emphasised the necessity for improved communication between multidisciplinary teams, with the aim to ensure that severe AS patients receive appropriate treatment.

**Methods/design:**

IMPULSE is a prospective, multicentre, European registry designed to gather data over 12 months on the treatment decisions made by referring physicians for patients newly diagnosed with severe AS. Each patient has a follow-up of 3 months. The study will consist of two observational phases to assess the appropriateness and rate of referral based on current guidelines prior to and after an interventional phase aiming to determine whether a simple quality of care intervention improves patient management.

**Discussion:**

Data will be analysed firstly, to determine the appropriateness of treatment decisions for the management of severe AS in current European clinical practice, and secondly, to evaluate the effectiveness of facilitated data relay from a designated echocardiography department nurse to the referring physician early after diagnosis in improving quality of care. Additionally, variables will be identified that are associated with inappropriate decision-making. Collectively, the aim will be to design a clinical pathway that will improve the timely management of patients with newly diagnosed severe AS.

## Background

Aortic stenosis (AS) is a common, serious valve disease with a prevalence that increases with age, affecting approximately 0.2% of patients aged 50–59, 1.3% of patients aged 60–69, 3.9% of patients aged 70–79, and 9.8% of patients aged 80–89 [1]. Of those aged over 75 years in Europe and North America, 3.4% have AS that is classed as severe [2], defined as a maximum jet velocity (Vmax) ≥4 m/s, an aortic valve area (AVA) ≤1.0 cm^2^, or a transvalvular pressure gradient ≥40 mmHg [3]. No effective medical treatment is available and if not treated by intervention (surgical aortic valve replacement (SAVR) or transcatheter aortic valve implantation (TAVI)), severe AS is associated with a rapid physical decline and a high rate of cardiac death within 3 years of symptom onset [4].

The standard means for diagnosis of severe AS is transthoracic echocardiography [5, 6]. ESC and ACC/AHA guidelines for subsequent management recommend that asymptomatic patients receive serial clinical assessment and an annual echocardiogram [3, 7]. Intervention (SAVR or TAVI) is indicated when patients develop symptoms or, if asymptomatic, when left ventricular ejection fraction (LVEF) is <50%.

Though SAVR significantly increases life expectancy and improves quality of life [8], a large proportion of patients with valvular disease have comorbid conditions that would place them at high risk during a surgical procedure. These factors include advanced age, poor LVEF, renal disease, and chronic obstructive pulmonary disease [9–12]. TAVI should be considered as an alternative in patients for whom surgery is contraindicated or estimated at high surgical risk, and data that are more recent suggest that TAVI may also be beneficial in intermediate-risk patients [13–16].

Despite the clear guidelines outlining the suitability and timing of these interventions [3, 7], several studies have reported non-adherence in clinical practice, with disparity between current scientific recommendations and treatment decisions estimated to occur in between 23 and 42% of AS patients [11, 17–19]. However, these studies of non-adherence in clinical practice mostly pre-date the availability of TAVI. Given the strong association of AS with short-term mortality, results showing inappropriate delay could highlight a need for improvement in the quality of care.

The main aims of the present study are firstly, to assess the rate and reasons for potential disparities between the proportion of patients diagnosed with severe AS and those actually treated, and secondly, to evaluate the impact of a simple, early, quality improvement intervention (in the form of facilitated data relay from a dedicated centre coordinator) to narrow the gap. Using this information, the aim of the study is to establish a clinical pathway to ensure adequate follow-up and management of AS patients.

## Methods/Design

IMPULSE is a prospective, international, multi-centre, registry-based cohort study evaluating the quality of care for patients with severe AS. The study combines observational and interventional elements in order to assess the current state of care and the potential for a simple intervention to improve quality. The aim is to enrol a study population of over 2500 patients consecutively across 25 centres in 10 European countries. The registry is established in accordance with the declaration of Helsinki (1964), and prior ethical agreements have been obtained from the appropriate ethics committee for each site. Patients are required to provide informed consent prior to enrolment, and data are collected by a co-ordinating study nurse in the form of electronic case report forms (eCRFs).

### Site selection

Only sites able to offer a comprehensive range of the currently available AS treatments will participate in the registry. A study nurse is employed at the centres specifically to monitor echocardiography results and to communicate with the referring physician (RP) in a structured manner.

### Patient selection

To be eligible for inclusion in the registry, patients must be aged 18 or over and diagnosed with severe AS by echocardiography. Severe AS is defined as having an aortic valve meeting at least one of the following criteria: 1) AVA < 1 cm^2^, 2) valve area index <0.6 cm/m^2^, 3) Vmax > 4.0 m/s, 4) mean transvalvular gradient > 40 mm Hg. All patients with previous AVR, or AS not considered severe according to the aforementioned criteria, will be excluded from the registry. Patients will be excluded if they have cognitive impairment that may limit their understanding of the factors involved in study participation, including the consent process. Enrolment will be consecutive.

### Data collection

All data are entered into the eCRF (s4trials, Berlin, Germany) and signed by the study nurse at the earliest opportunity, using registry identification numbers rather than names to protect identity. Automatic checks for plausibility and completeness are performed, and all data sets will be examined for irregularities by the data manager.

For each patient, baseline characteristics (including symptoms, co-morbidities, frailty, and EuroSCORE values), echocardiogram findings, and contact details will be documented at the point of individual study enrolment (where enrolment is defined as the time of informed consent directly after echocardiography-based diagnosis of severe AS). In addition, symptom status, co-morbidity, frailty, and eligibility for different treatment options (pharmaceutical management, SAVR, or TAVI) will be collected by the study nurse.

The main follow-up will be performed for all patients via a structured telephone call or contact with the RP 3 months after the date of each individual enrolment (i.e. a patient diagnosed and enrolled on day 9 will be followed-up at 3 months + 9 days). Follow-up data will include vital status, emergency admissions, referrals, and treatment since enrolment, as well as the sub-specialty of the healthcare professional responsible for making such a decision. Vital status at one year will be also collected via chart review and contact of the RP.

For the subset of patients included in the interventional phase only (see definitions below), data regarding the vital status of a particular patient, and the treatment plans or referral intentions of the RP (active decision not to treat, watchful waiting, conservative management, balloon aortic valvuloplasty (BAV), SAVR, or TAVI) will be obtained 1 week after the date of each individual enrolment. Watchful waiting is defined as the scheduling of a further outpatient assessment, stress echocardiography, exercise testing, transoesophageal echocardiography, catheterisation, computed tomography, or referral to a valve service or other clinician. Conservative management is defined as medical therapy only.

### Study phases and interventions

**Fig. 1:**
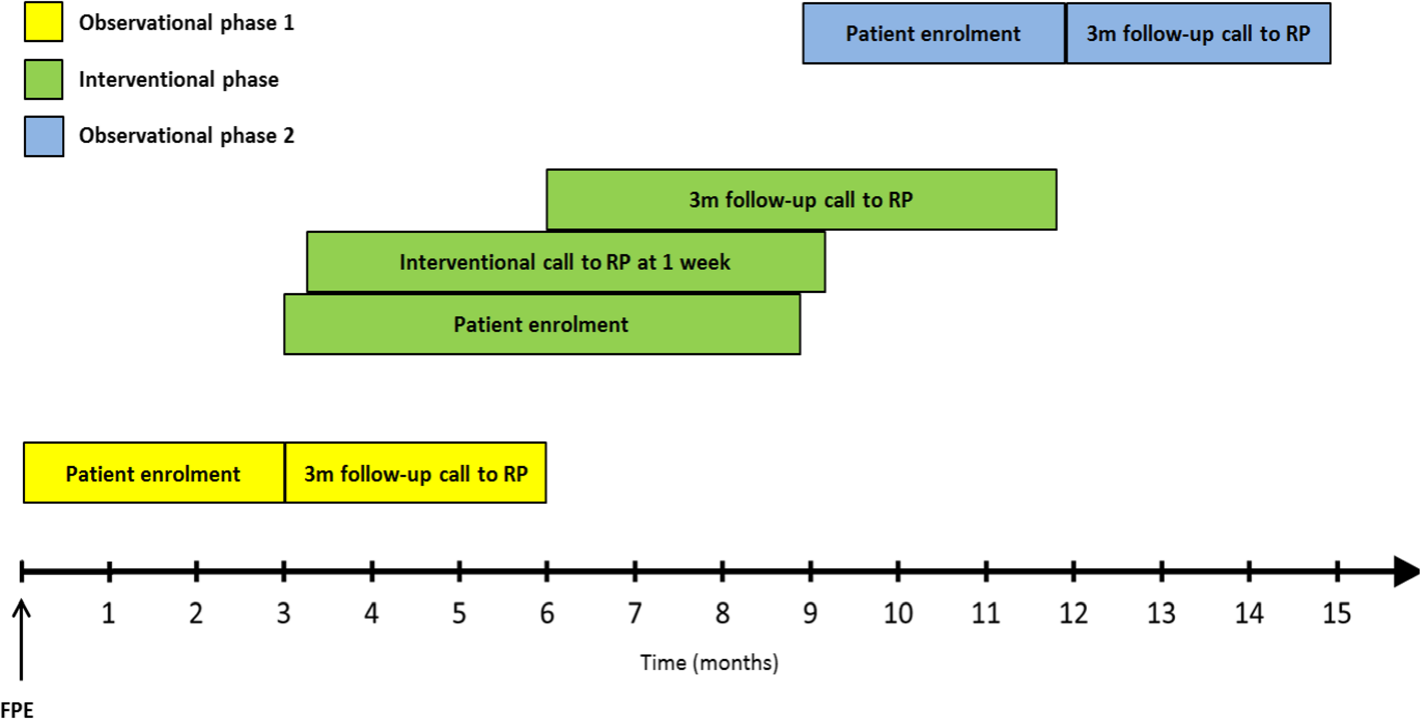
Timeline of the IMPULSE Study. This timeline refers to the study progression at each individual site, starting from enrolment of the first patient at that site. Each bar is indicative of the upper and lower limits of the period within which any one event occurred. Accordingly, follow-up and interventional call timings are relative to the point of individual patient enrolment (i.e. a patient diagnosed and enrolled on day 9 will be followed-up at 3 months + 9 days). FPE: First Patient Enrolled at the particular site; RP: Referring Physician

Study duration will be 15 months at each participating site, starting from the point of first patient enrolment. This study period is divided into 3 distinct phases (Fig. 1) with 3 different patient groups, as follows:

- **Observational phase 1:** Includes all patients enrolled between month 0 and month 3. No contact is made with the RP regarding the IMPULSE study until follow-up at 3 months, at which time details on medical treatment in the interim are collected.
- **Interventional phase:** Includes all patients enrolled between month 4 and month 9. The RP is contacted by the study nurse 1 week after individual patient enrolment and advised that their patient has been diagnosed with severe AS (facilitated data relay) and consented to participation in the present quality improvement study. The RP is asked to provide details on the vital status of the patient and their intended treatment plans at this point. They are then contacted again at 3 months; at which time, details on treatment in the interim are collected.
- **Observational phase 2:** Includes all patients enrolled between months 10 and 12. This phase is carried out as the observational phase 1 to adjust for potential seasonal variation in the clinical presentation of the patients and with the intention of detecting any persisting influence of the interventional phase on the subsequent treatment decisions made by RPs.

### Outcome measures

The primary outcome measure is the rate of SAVR or TAVI by the RP at 3-month follow-up in each of the 3 phases. Secondary outcome measures are as follows: 1) the rate of referral for exercise testing or stress echocardiography by the RP at 3 month follow-up, 2) time to next out-patient appointment or clinical review, 3) type of treatment plan selected (active decision not to treat, watchful waiting, conservative management, BAV, SAVR, or TAVI), 4) the appropriateness of treatment decisions made in collaboration with the valve specialist, and 5) survival outcome one year post-diagnosis.

### Statistics

We estimated that we would be able to capture a mean of 100 consecutive patients per site, 25 during the first observational phase, 50 in the interventional phase and another 25 during the second observational phase. The final sample size estimate was based on the aim to involve 25 sites in different countries and health care systems across Europe and to ask them to enrol patients throughout a year.

Intention-to-treat analysis including all enrolled patients will be carried out. Descriptive summaries will be used to present the collected evaluation data. For categorical variables (e.g. gender) frequency distributions will be given. Statistical significance will be determined using a chi-squared or a Fisher’s exact test. For numeric variables (e.g. patient age) minimum, maximum, mean, median, and standard deviation will be calculated. Statistical significance will be determined using a student’s *t*-test in the case of normally distributed data, or a Mann–Whitney *U*-test for non-normally distributed data. Linearized rates and actuarial probability statistics may be used where appropriate for adverse event reporting. Kaplan-Meier analysis will be performed for survival outcomes and, where appropriate, for adverse event outcomes.

## Discussion

In the present multinational, registry-based, prospective analysis, we aim to gather data on the appropriateness of current follow-up and referral after diagnosis of severe AS in Europe, and further establish the impact of a simple quality improvement intervention on this practice. Using the considerable quantity of information gathered, this should allow us to establish an evidence-based clinical pathway for the effective management of patients with severe AS.

### Current practice

In the first observational phase, the appropriateness of treatment decisions for the management of severe AS in current European clinical practice will be evaluated, and the scale of any deficit quantified. This is following several previous studies that have revealed consistently low rates of appropriate management in a considerable proportion of AS patients. In 2003, the large-scale Euro Heart Survey showed that 31.8% of patients with severe, symptomatic, single-valvular heart disease did not receive an intervention, and that surgery was denied in one third of AS symptomatic patients [20]. Later, in 2010, Freed et al. reported from a small study in 106 patients, that 69% of severe AS patients were not referred for valve replacement, of which 42% were symptomatic and 21% had LVEF <50%; therefore making them eligible for consideration under ACC/AHA guidelines [18]. More recent data have reported similar failings in monitoring valve disease, with a large but retrospective series identifying that only 59% of patients with moderate-severe left-sided valve disease received follow-up echocardiography within 60 days of the time recommended in existing guidelines [17]. Therefore, a deficit between eligibility and adequate management has been documented consistently, regardless of period, country, disease severity, or available treatment.

A combination of patient and physician-based factors has been proposed to explain the gap between guidelines and clinical practice [17]. Clinicians in the Euro Heart Survey reported advanced age, severe left ventricular dysfunction, recent myocardial infarction, severe coronary disease, prior neurological disease, and a decrease in symptoms under medical treatment as justifications for not referring a patient for SAVR [20] but several of these potential reasons are highly debatable. Additionally, Freed et al. found that the lack of referral for SAVR or TAVI in symptomatic patients was mainly due to failure of the treating physician to attribute symptoms to AS (29%), followed by an elevated surgical risk (15%), despite 40% of the latter patients having low EuroSCORE parameters [18]. Furthermore, existing methods for determining surgical risk (such as the logistic EuroSCORE model and on-line STS Risk Calculator) have many pitfalls and limitations, and appear not to reliably predict the true likelihood of mortality in all patients; particularly those undergoing TAVI [21]. Thus, a RP with poor knowledge of the limitations of current assessment tools in valvular heart disease may erroneously prevent eligible patients from undergoing lifesaving intervention.

Taken together, these results suggest that appropriate medical attention may be delayed or withheld in a significant proportion of patients due to poor decisions made by RPs acting independently rather than in the environment of a ‘heart team’ discussion. Observational phase 1 of the present study will supply further insight into the validity of this assumption in contemporary practice, and provide data on the type of physician currently responsible for making treatment decisions. Furthermore, justifications for non-referral will be collected to identify variables associated with inadequate treatment decisions. This may be useful for educating physicians and improving appropriateness of future treatment.

### Quality improvement intervention

Several different strategies for quality improvement have been suggested to enhance patient care. The need for a well-organised valve service to improve monitoring, evaluation, and timing of intervention for patients with AS has been highlighted in several recent publications [22–24]. A key element of this valve service is to ensure effective, multidisciplinary communication between team members, including echocardiographers and clinicians, co-ordinators, interventional cardiologists, and cardiothoracic surgeons [23]. This should, in theory, maximise the number of patients receiving the appropriate therapy, increase referral rates for intervention and thereby minimise morbidity and mortality. However, with cost effectiveness always a key concern, healthcare institutions favour economical interventions with a clear business case [22].

A previous study by Taggu et al. described the positive outcomes of a quality of care intervention for patients with valvular disease, reporting that the agreement between clinical follow-up and best practice guidelines rose by 51% following the establishment of sonographer-led valve clinics for patients with stable valve disease [24]. This highlights the capacity for improvement in the care of patients with valvular disease, although main focus was on monitoring rather than on intervening. In the interventional phase of the present study, the effect of employing a single, dedicated echocardiography clinic nurse to notify the RP of a finding of severe AS will be evaluated. Outcomes such as referral rate will be compared to those seen in observational phase 1 to detect any improvements. This type of intervention via a phone call or other non-automated method is known as facilitated relay of patient data [25]. In a previous, large systematic review of quality improvement strategies in the care of hypertensive patients, implementation of simple facilitated data relay was shown to reduce systolic and diastolic blood pressure by a median of 8.0 and 1.8 mmHg, respectively, and result in 25.1% more patients attaining systolic blood pressure within their target range [26]. This suggests that such an intervention can have a substantial impact on patient care, though it has never been tested in the context of severe AS. Should the present study demonstrate significant improvements in the appropriateness of patient referral for timely and appropriate intervention under the facilitated data relay intervention, implementation would be simple and low-cost, making it an attractive strategy for improving quality of care.

For the third phase of the present study, the facilitated relay of diagnosis will be stopped and its legacy effect on patient management evaluated. This will indicate whether the intervention was efficient at changing the behaviour of the RPs and indicate the potential for short interventions to be effective in the longer-term.

### Potential limitations

Firstly, the largely observational nature of the IMPULSE study means that investigators will have little control over the characteristics of the severe AS patients enrolled. Considering that 3 distinct populations will be used for different phases of the study, this may result in an uneven distribution of treatment decisions between phase groups. Accordingly, the appropriateness of RP decisions may reflect mistakes due to more challenging patient variables, rather than active deviations from guidelines. However, this is unavoidable if a snapshot of real-world practice is to be obtained, and the high patient numbers planned for enrolment over a reasonable time period (minimum 3 months) should accommodate for such imbalances.

The study does not involve an investigation into the contribution of patient/family preferences to the treatment decision-making process. It will therefore not be possible to exclude this important variable from the assessment of the appropriateness of the RP’s decision. In the present analysis we will focus on evaluating guideline adherence, with future work potentially delving into the underlying reasons for RPs deviating from the recommendations.

The relatively short study duration means that guideline adherence will only be evaluated up to 3 months post-diagnosis. This means that long-term consequences of appropriate/inappropriate management are beyond the scope of the current study. Additionally, phase 3 will only be suitable for assessing the short-term legacy of the quality of care intervention, and further studies will be necessary to establish any longer-term effects. However, for providing an initial view into the efficacy of facilitated data relay to improve initial care decisions in severe AS patients, 3 month follow-up is sufficient.

Lastly, during the facilitated data relay intervention stage, the physician is not only informed of diagnosis outcomes, but also of their patient’s participation in a study. This may result in a feeling that their professional capacity is being evaluated, prompting more conscientious decision-making. Although clearly an advantage for study participants, this may result in over-estimation of the effect the quality of care intervention would have under real-world, non-study conditions. However, in order to comply with ethical guidelines, such a notification is unavoidable.

## Conclusion

This large-cohort, prospective, multinational, registry-based study will generate important data regarding current rates of good practice guideline adherence in patients diagnosed with severe AS, and the potential for a simple quality of care intervention in the form of facilitated data relay to improve this. This information will be used to establish a clinical pathway that can be applied to healthcare services to ensure appropriate patient management. Accordingly, IMPULSE may contribute to reductions in morbidity and mortality rates associated with the referral gap in severe AS patients.

## Abbreviations

ACC: American College of cardiology
AHA: American heart association
AS: Aortic stenosis
AVA: Aortic valve area
BAV: Balloon aortic valvuloplasty
eCRF: Electronic case report form
ESC: European society of cardiology
LVEF: Left ventricular ejection fraction
RP: Referring physician
SAVR: Surgical aortic valve replacement
TAVI: Transcatheter aortic valve implantation
Vmax: Maximum jet velocity
